# Boiling histotripsy lesion characterization on a clinical magnetic resonance imaging-guided high intensity focused ultrasound system

**DOI:** 10.1371/journal.pone.0173867

**Published:** 2017-03-16

**Authors:** Avinash Eranki, Navid Farr, Ari Partanen, Karun V. Sharma, Hong Chen, Christopher T. Rossi, Satya V. V. N. Kothapalli, Matthew Oetgen, AeRang Kim, Ayele H. Negussie, David Woods, Bradford J. Wood, Peter C. W. Kim, Pavel S. Yarmolenko

**Affiliations:** 1 Sheikh Zayed Institute for Pediatric Surgical Innovation, Children’s National Health System, Washington DC, United States of America; 2 Center for Interventional Oncology, Radiology and Imaging Sciences, Clinical Center, National Institutes of Health, Bethesda, Maryland, United States of America; 3 Clinical Science MR Therapy, Philips, Andover, Massachusetts, United States of America; 4 Department of Biomedical Engineering, School of Engineering & Applied Science, Washington University, St. Louis, Missouri, United States of America; Michigan State University, UNITED STATES

## Abstract

**Purpose:**

High intensity focused ultrasound (HIFU) is a non-invasive therapeutic technique that can thermally ablate tumors. Boiling histotripsy (BH) is a HIFU approach that can emulsify tissue in a few milliseconds. Lesion volume and temperature effects for different BH sonication parameters are currently not well characterized. In this work, lesion volume, temperature distribution, and area of lethal thermal dose were characterized for varying BH sonication parameters in tissue-mimicking phantoms (TMP) and demonstrated in *ex vivo* tissues.

**Methods:**

The following BH sonication parameters were varied using a clinical MR-HIFU system (Sonalleve V2, Philips, Vantaa, Finland): acoustic power, number of cycles/pulse, total sonication time, and pulse repetition frequency (PRF). A 3×3×3 pattern was sonicated inside TMP’s and *ex vivo* tissues. Post sonication, lesion volumes were quantified using 3D ultrasonography and temperature and thermal dose distributions were analyzed offline. *Ex vivo* tissues were sectioned and stained with H&E post sonication to assess tissue damage.

**Results:**

Significant increase in lesion volume was observed while increasing the number of cycles/pulse and PRF. Other sonication parameters had no significant effect on lesion volume. Temperature full width at half maximum at the end of sonication increased significantly with all parameters except total sonication time. Positive correlation was also found between lethal thermal dose and lesion volume for all parameters except number of cycles/pulse. Gross pathology of *ex vivo* tissues post sonication displayed either completely or partially damaged tissue at the focal region. Surrounding tissues presented sharp boundaries, with little or no structural damage to adjacent critical structures such as bile duct and nerves.

**Conclusion:**

Our characterization of effects of HIFU sonication parameters on the resulting lesion demonstrates the ability to control lesion morphologic and thermal characteristics with a clinical MR-HIFU system in TMP’s and *ex vivo* tissues. We demonstrate that this system can produce spatially precise lesions in both phantoms and *ex vivo* tissues. The results provide guidance on a preliminary set of BH sonication parameters for this system, with a potential to facilitate BH translation to the clinic.

## Introduction

The current standard of care in treatment of benign and malignant tumors involves a multimodal approach [1]. While most oncology approaches focus on systemic therapies, local and regional control may be important in the case of oligometastatic disease, locally contained disease, and potential future combinations with systemic immunotherapies such as checkpoint inhibitors. Options for local treatment include both invasive surgical approaches and minimally invasive therapies. Minimally invasive therapies include radiofrequency (RF) [2, 3], cryo [4], microwave (MW) [5], and laser ablation [6, 7], and percutaneous ethanol injection [8, 9]. However, even minimally invasive techniques risk collateral tissue damage and procedure-related complications [10–14]. Treatment approaches that are effective, non-invasive, spatially precise, and relatively quick to perform have the potential to improve management of local disease by reducing pain, risk of infection, collateral damage to intervening and surrounding tissues, and overall hospital costs.

One non-invasive technique that can address shortcomings of invasive approaches for local therapy is high intensity focused ultrasound (HIFU). HIFU uses acoustic waves to precisely focus ultrasound energy within the body. Deposition of this ultrasound energy may have mechanical and thermal effects. Thermal ablation (>60°C) increases tissue temperature at the focal zone and has been the most common method of tissue destruction using HIFU [15]. This approach has been used to treat a wide variety of tumors including uterine fibroids [16, 17], liver tumors [18, 19], kidney tumors [18, 20], bone tumors [21, 22], and prostate cancer [23, 24]. In addition to thermal ablation, HIFU has also been used to achieve mild hyperthermia (40–45°C) [25, 26] and to cause mechanical destruction of tissue, also known as histotripsy [27]. Magnetic resonance imaging (MRI) [28–30] and ultrasound imaging [31, 32] are currently used in planning, guiding, and monitoring HIFU therapies. Despite real-time monitoring methods such as MRI thermometry, HIFU thermal ablation has limitations including near field heating or collateral damage of tissue adjacent to the treated region that may limit its clinical applicability [33]. While MR-thermometry may provide both therapy monitoring and closed-loop feedback control [34], heat diffusion and off-target heating may cause iatrogenic peri-target tissue injury [24, 35]. Furthermore, ablative temperatures may not be easily attainable in highly perfused organs without damage to intervening tissues. Treatment approaches that do not entirely rely on temperature rise may thus circumvent certain limitations of HIFU thermal ablation.

Recently, HIFU methods termed cavitation histotripsy [36, 37] and boiling histotripsy (BH) [38, 39] have been used to create mechanical disruption of tissue. Unlike traditional HIFU thermal ablation that typically uses continuous wave or high duty cycle pulsed wave ultrasound, histotripsy employs pulsing regimes at much lower duty cycles (even lower than conventional 10–20% duty cycle “pulsed HIFU”). Cavitation histotripsy fractionates tissue via a dense bubble cloud, created using microsecond-long pulses at high pulse repetition frequency (PRF). This technique has been demonstrated *in vitro* and *in vivo* for a variety of medical applications [36, 40–42]. However, cavitation histotripsy has several challenges such as unexpected interruption of cavitation activity during the course of treatment due to the stochastic behavior [37]. It is therefore important to develop histotripsy methods that are more predictable and controllable.

BH is a more predictable technique compared to cavitation histotripsy [38]. BH generates a millimeter-size boiling bubble, causing near instantaneous tissue emulsification. Unlike cavitation histotripsy, BH employs millisecond-long pulses, with lower PRF. In this method, high-amplitude acoustic wave creates a boiling bubble at the focus. This wave also produces shock fronts consisting of several high order harmonics of the fundamental frequency, causing increased absorption of energy and further enhancing heating to approximately 100°C in milliseconds. This highly localized explosive boiling and its further interaction with subsequent shock fronts results in instant tissue death. Since this explosive boiling takes place in the order of milliseconds, the impact of heat accumulation is minimal and results in negligible collateral thermal injury, thereby potentially allowing for greater spatial precision of the treatment.

Currently, no clinical HIFU system has been characterized to accurately study the effects of various BH parameters, an important step to facilitate translation of BH to the clinic. The purpose of this study was to characterize lesion volume, temperature distribution, and thermal dose using BH delivered by a commercially available clinical MRI-guided HIFU system (MR-HIFU) in both tissue-mimicking gel phantoms and in *ex vivo* porcine liver and cardiac muscle.

## Methods and materials

### Experimental setup

A clinical HIFU system (Sonalleve V2, Philips, Vantaa, Finland) integrated with a MRI scanner (Achieva 1.5T, Philips, Vantaa, Finland) was used to perform the BH experiments. The system is capable of accurately delivering acoustic energy, and consists of a generator cabinet and a patient tabletop that houses an ultrasound transducer attached to a positioning system with 5 degrees of freedom, submerged in a sealed oil tank. Both the transducer positioning and the generators are controlled using a dedicated therapy-planning console. The transducer is a 256-element spherical-shell phased-array operating at 1.2 MHz and with a focal length of 14 cm. The ultrasound beam propagates through a sealed acoustically hypoechoic window and produces an ellipsoidal focal point of approximately 1.6 × 1.6 × 10 mm in size (-6 dB of positive pressure) [26]. The MR-HIFU system also includes MRI receive coils, consisting of two integrated elements within the tabletop and a three-element pelvic coil.

**Fig 1:**
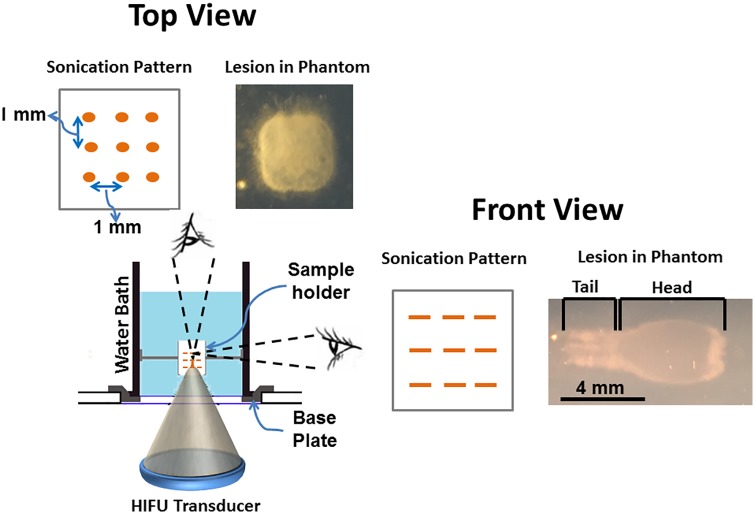
Diagram showing the experimental setup used to produce BH lesions on a clinical MR-HIFU system in both tissue-mimicking phantom and ex vivo tissue. The setup consists of a water bath, filled with degassed water. This water bath was placed on an acoustically hypoehoic membrane. This membrane made it possible for the HIFU beam to pass through and into the water bath. The sonication pattern is a 3 × 3 × 3 cube with 1 mm spacing between each point in either direction. The lesion in top-view, looks like a square, and appears ‘tad-pole’ shaped in any plane parallel to the HIFU beam.

The overall arrangement used for the BH experiment is depicted in Fig 1. A cylindrical water bath filled with deionized and degassed water at room temperature (23°C) and sealed with an acoustically hypoechoic Mylar membrane on one end was placed on an acrylic base-plate designed to position the water bath over the acoustic window. A custom, 3D-printed phantom holder was designed to position a phantom within the water bath. The holder consisted of a plastic box with openings at the bottom and top surfaces to facilitate sonication and exiting beam path. The phantom material was inserted into this plastic holder, and removed after each experiment. An acoustic absorber pad was placed 2 cm from the top of the phantom holder to prevent reflections within the water bath.

### Hydrophone measurements

Hydrophone measurements were made across a range of power levels to assess changes in acoustic pressures. A custom-made water tank with an inner diameter of 260 mm and a height of 610 mm was placed on top of the patient table and filled with deionized and degassed water. A fiber-optic hydrophone (HFO-690, Onda Corporation, Sunnyvale, CA, USA) was attached to a 3-D positioner (Velmex Inc., Bloomfield, NY, USA). A custom MATLAB (MathWorks, Natick, MA, USA) program was used to control the 3-D positioner, acquire hydrophone signals using a digitizer (Gage Applied Technologies Inc., Lockport, IL, USA), synchronize data acquisition, and process the acquired data. The HIFU system was controlled to generate ultrasound pulses with different acoustic powers (500, 550, 600, or 650 W) at 1.2 MHz frequency, 10 Hz PRF, and 40 cycles/pulse. The lower power threshold was selected based on the ability to produce repeatable lesions at the lowest acoustic power, while the higher power threshold was selected to ensure the continuous safe operation of the HIFU transducer. The 3-D positioner was controlled to locate the HIFU focus, and three repeated pressure measurements were performed at the focus for each power value to obtain average peak positive and peak negative pressure values.

### Tissue mimicking phantom & *ex vivo* tissue preparation

Tissue-mimicking phantoms for *in vitro* histotripsy lesion volume characterization experiments were prepared as previously reported [43–45]. Briefly, a commercially available aqueous solution of acrylamide-bisacrylamide (40% w/v) with a feed ratio of acrylamide/ bisacrylamide 19:1, was mixed with degassed water and placed in a vacuum chamber for 15–20 minutes. Then, the solution was mixed with tetramethylethylenediamine (TEMED) and ammonium persulphate (APS) solution, immediately transferred to rectangular containers (6 × 6 × 10 cm) to match the phantom holder size, and left to cure at room temperature. The density of this phantom material was measured to be 1.0200 ± 0.0014 g/cm^3^ using a commercially available apparatus (density kit for XS precision balances, Mettler Toledo, Columbia, MD, USA), i.e., within the range of healthy human liver and spleen tissues [46]. Additionally, the formulation of our phantom is similar to the one reported previously by Zell *et al*. [47]. Therefore, the attenuation and speed of sound of our tissue mimicking phantom closely matches 0.7 ± 0.1 db/cm at 5MHz and 1.58 ± 0.03 × 10^3^ m/s respectively.

Tissues were obtained after euthanasia of a healthy adult 150 kg pig on an unrelated Animal Care and Use Committee approved protocol of the National Institutes of Health. Liver and cardiac muscle tissues were obtained within one hour and samples immediately prepared to fit the tissue holder. These tissues were selected due to their diverse structural organization and biochemical composition. Values of attenuation of the liver and cardiac muscle tissue were derived from literature for calculations (0.676 and 0.8 dB/cm/MHz, respectively) [48]. The prepared tissues were transported in a bag of phosphate buffered saline (PBS, 1x) on ice. This approach retains tissue function and viability for up to three days [49]. All tissue samples were degassed for two hours in a vacuum desiccant chamber. Once degassed, the tissues were again placed in PBS on ice and transported to the MRI suite.

### MRI treatment planning and monitoring

After positioning the sample and absorber pad within the water bath and strapping the pelvic coil over the bath, a survey scan was performed to localize the phantom (Turbo Field Echo (TFE) 3D; field of view (FOV): 300 × 300 × 120 mm^3^; voxel Size: 1.46 × 1.73 × 12 mm^3^; stacks: 2; number of slices 12/5). This scan was followed by susceptibility-sensitive 3D steady-state fast-field-echo (FFE) sequence; repetition time (TR)/echo time (TE): 150/15 ms; FOV: 280 × 280 × 25 mm^3^; voxel size: 1.2 × 1.2 × 2.5 mm^3^; acquisition time: 76 seconds) to check for air bubbles in the ultrasound beam path. HIFU exposures were planned on a T2-weighted image set acquired using an FFE pulse sequence; TR/TE: 680/35 ms; flip angle (FA): 20°; FOV: 250 × 250 × 75 mm^3^; voxel size: 1.2 × 1.3 × 1.5 mm^3^; parallel imaging (SENSE) factor: 2 (in RL direction); orientation: coronal; slices 20; acquisition time: 12 min). T1W image set was acquired prior to start of the sonication (number of signal averages (NSA) = 2; 3D FFE; FOV: 200 × 250 × 81 mm^3^; Voxel size: 1.3 × 1.5 × 1.3 mm^3^; TR/TE: 20/4.6 ms). The MRI temperature mapping sequence was a 2D echo-planar FFE (FFE-EPI) pulse sequence (TR/TE: 36/19 ms; EPI factor = 11; FA = 20°; FOV = 160 × 121 mm^2^; voxel size = 2.5 × 2.5 × 7 mm^3^; 4 slices: 3 coronal and 1 sagittal; dynamic scan time = 1.8 s. Temperature and thermal dose maps were calculated in real-time using the MRI phase images and the proton resonance frequency shift (PRFS) thermometry method [50], overlaid over the magnitude images, and displayed on the therapy planning console. For the *ex vivo* experiments, the T2W and T1W MRI sequences were repeated post HIFU to visualize and characterize the lesions.

### HIFU sonication parameters

Sonication planning was performed on the therapy planning console based on MR images. A location 30 mm deep within the phantom or tissue was selected to produce a pattern consisting of 27 locations spatially separated by 1 mm (in between each focal point), in a 3 × 3 × 3 grid. A schematic figure of planned sonication pattern is shown in Fig 1. To relate the experiments conducted at room temperature to *in vivo* studies, reference temperature for MR-thermometry was set to 37.5°C, and temperature was calculated relative to this baseline temperature. The sonication parameters used in TMP’s are detailed in Table 1.

**Table 1:** List of all sonication parameters used in BH characterization experiments. Four sonication parameters were selected for this experimental study. While each of these parameters are varied (in bold font), other three parameters are kept constant.

Acoustic Power (W)	Number of cycles/pulse	Pulse Length (ms)	Total Sonication Time (seconds)	Pulse Repetition Frequency (Hz)
**500**	15,000	12.5	902	1
**550**				
**600**				
**650**				
600	**10,000**	**8.3**	~902	1
	**12,000**	**10**		
	**14,000**	**11.6**		
	**16,000**	**13.3**		
	**18,000**	**15**		
	**20,000**	**16.6**		
600	15,000	12.5	**137**	1
			**274**	
			**410**	
			**574**	
			**684**	
			**820**	
600	16,000	13.3	800	**0.5 (0.66% DC**^a^**)**
			400	**1 (1.33% DC)**
			200	**2 (2.66% DC)**
			133	**3 (4.00% DC)**
			100	**4 (5.33% DC)**
			80	**5 (6.66% DC)**

^a^ DC = Duty Cycle. The cube was sonicated 15 times while varying PRF.

*Ex vivo* porcine liver and cardiac tissues were sonicated with the following parameters:

1. 5 Hz PRF, 600 W and 15,000 cycles/pulse
2. 1 Hz PRF, 600 W and 20,000 cycles/pulse

### Estimating time-to-boil

The parameter time-to-boil was estimated for all acoustic powers applied in our experiments. The ultrasound waves at the focus induced heating, the rate of which can be calculated using weak shock theory [51]:

$$H=\;\frac{\beta f_{o}A_{s}^{3}}{6\rho_{o}^{2}c_{o}^{4}}$$

where *H* is the heating rate, *f*_*o*_ is the ultrasound frequency, *A*_*s*_ is the in-situ shock amplitude, *ρ*_*o*_ is the density of the medium and *c*_*o*_ is the speed of sound. If the heating rate is sufficiently high, the effect of heat conduction to surrounding regions can be low and neglected. Using the heating rate, the time-to-boil can be calculated as:

$$t_{b}=\;\frac{\Delta Tc_{v}}{H}$$

where Δ*T* is the difference between 100°C and local temperature and *c*_*v*_ is the heat capacity/volume. We used the following constants to calculate both heating rate and time-to-boil in the tissue-mimicking phantom: *β* = 4, *f*_*o*_ = 1.2 MHz, *ρ*_*o*_ = 1.02 g/cm^3^, *c*_*o*_ = 1544 m/s, *c*_*v*_ = 5.3 × 10^6^ J/m^3^°C.

### 3D lesion segmentation

Post sonication, the lesions were individually scanned using a clinical diagnostic ultrasound scanner (iU22, Philips, Bothell, WA, USA) equipped with a 3D transducer (X6-1) with elements arranged in a matrix array, operating at 6 MHz. All phantoms were oriented along the same direction with respect to the transducer during scanning. An acoustic absorber pad was placed under each phantom to prevent reverberation. Post scanning, 3D DICOM images of the lesions were stored for further analysis, and the phantoms were bisected along the beam axis (MRI sagittal plane) to observe gross damage. A semi-automatic segmentation software (TurtleSeg, The University of British Columbia, Canada) based on gradient magnitude, gradient direction, and Canny edge detection, was used to obtain lesion volumes [52, 53]. Briefly, sparse number of slices along the sagittal plane was manually contoured and the software was able to automatically connect the remaining slices to provide a lesion volume estimate.

### MRI temperature & thermal dose data analysis

The MRI temperature maps were analyzed using MATLAB (MathWorks, Natick, MA, USA). A 40 × 30 mm region-of-interest (ROI) was centered on the targeted region and the maximum temperature values within this ROI were obtained over the entire duration of the sonication. Full width at half maximum (FWHM) of temperature elevation was calculated as a standardized quantitative measure of temperature distribution within the ROI. Cumulative equivalent minutes at 43°C (CEM43) was used as a metric for thermal dose assessment since it correlates well with thermal tissue damage [54, 55]. Area of thermal dose >240 CEM43 was computed for every sonication in tissue-mimicking phantom.

### Histology

Post sonication, the tissues were fixed in 10% neutral buffered formalin for histological processing. Tissues were embedded with paraffin and sectioned perpendicular to the HIFU beam path, into 5μm thick slices. Subsequently, tissues were stained with hematoxylin and eosin (H&E) and imaged at 4x, 10x, and 40x magnification (Olympus BX 51-P, Waltham, MA, USA).

### Statistical analysis

Quantitative results were reported as mean ± standard deviation (SD) for all experiments conducted on tissue mimicking phantoms (N = 3 each sonication parameter). Comparisons amongst parameter groups were performed using one-way ANOVA subject to Bonferroni correction using GraphPad Prism (Version 5.01, GraphPad Software Inc., La Jolla, CA). For all tests, two-tailed p-values were obtained, and differences were considered significant if p≤0.05. Correlation was performed between variables and Pearson’s coefficient was computed.

## Results

### HIFU field assessment

**Fig 2:**
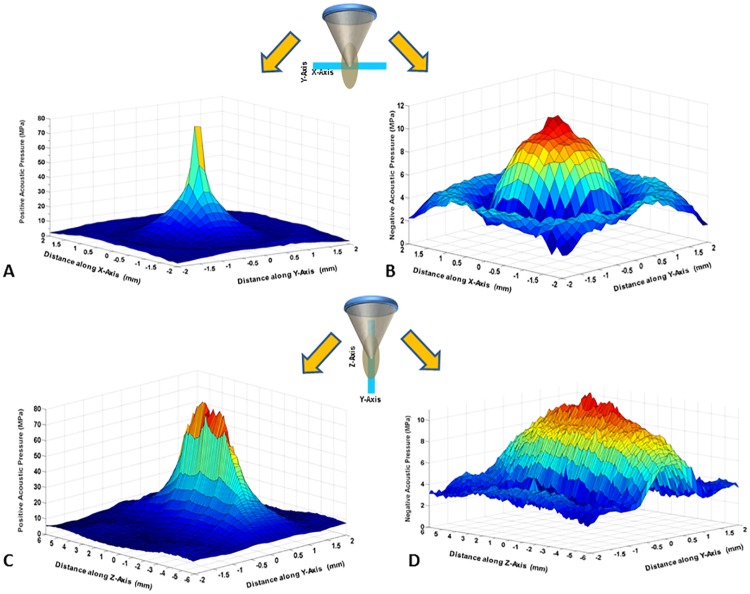
HIFU positive and negative pressure waveforms displayed in 3D at 500 W (free-field), across (A & B) and along (C & D) the plane of the HIFU beam propagation. At this acoustic power, the peak positive pressure was 65.97±4.42 and peak negative pressure of 10.98±0.45. Additionally, the beam width along the HIFU beam propagation is greater than the across the HIFU beam-axis.

Acoustic pressures were measured at four acoustic power levels. Increasing acoustic power from 500 W to 650 W increased peak positive pressure from 65.97 to 76.26 MPa and reduced peak negative pressure from -10.98 to -12.16 MPa (Fig 2, Table 2). High-pressure shock fronts become more noticeable with increasing power. This in turn reduced the time-to-boil, causing quicker heating and breakdown of tissue. Increase in these shock fronts also enhances the heating rate [51].

**Table 2:** Peak positive and negative pressures measured at varying acoustic powers using a fiber optic hydrophone.

Acoustic Power (W)	Peak Positive Pressure (MPa)	Negative Positive Pressure (MPa)
500	65.97±4.42	10.98±0.45
550	70.20±4.81	10.65±0.86
600	72.65±4.53	10.98±0.76
650	76.26±5.67	12.16±0.70

### a. Effect of BH sonication parameters on lesion volume

Sonications were performed while varying parameters (Table 1) to examine their effects on lesion volume in tissue-mimicking phantoms and in *ex vivo* tissue. The time-to-boil was estimated to be 5.45, 4.70, 4.25, and 3.59 ms for acoustic powers of 500, 550, 600, and 650 W, respectively. Resulting lesions were mechanically fractionated and had a characteristic ‘tadpole’ shape with all tested parameters as seen in Fig 1. Lesions created with sonication parameters greater than 12,000 cycles/pulse, 550 W, 1 Hz, and 274 seconds resulted in a void filled with semi-solid phantom debris at the focal zone, with little or no fractionation outside this region. Other parameters resulted in minimal fractionation of the phantom material at the focal zone, also with little or no damage outside this region.

#### Acoustic power

**Fig 3:**
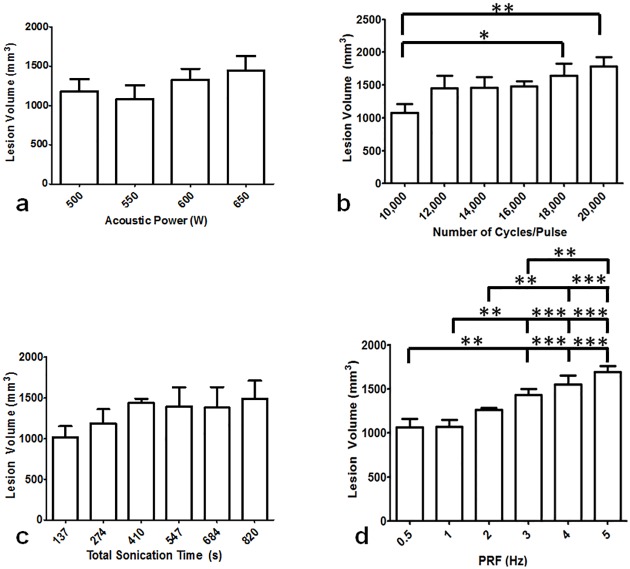
Lesion volume in tissue-mimicking phantom for varying BH sonication parameters. **a**. Lesion volume did not vary significantly for acoustic power 500–650 W at constant PRF of 1 Hz (p>0.05, ANOVA). **b**. Lesion volume with varying cycles/pulse. Significant differences (p ≤ 0.05) in lesion volumes were found between 10,000 and 18,000 cycles/pulse as well as 10,000 and 20,000 cycles/pulse. **c**. Varying total sonication time between 137 to 820 seconds resulted in no significant differences (p ≤ 0.05). **d**. Pulse repetition frequency (PRF) was varied from 0.5 to 5 Hz. The lesion volumes were significantly different between 0.5 and 3, 4, and 5 Hz (p ≤ 0.05). Similar differences were found between 1 and 3, 4 and 5 Hz, as well as between 2 and 4 and 5 Hz (p ≤ 0.05).

Upon gross visual analysis, the lesions appeared to increase in size with increasing acoustic power. Based on 3D ultrasound data, the average lesion volume increased linearly (R^2^ = 0.71), from 1180 ± 150 mm^3^ to 1440 ± 180 mm^3^ as acoustic power increased from 500 to 650 W (Fig 3a), though one-way ANOVA revealed no significant differences in lesion volumes (overall ANOVA p = 0.106).

#### Number of cycles/pulse

Across the studied range of cycles/pulse, linear increase (R^2^ = 0.86) in lesion volume was obtained with overall significant difference (overall ANOVA p = 0.0025, Fig 3b). Lesion volume (1080 ± 140 mm^3^) obtained at 10,000 cycles/pulse differed significantly from volumes obtained at both 18,000 and 20,000 cycles/pulse (1640 ± 180 mm^3^ and 1780 ± 140 mm^3^, p ≤ 0.05 and p≤0.01, respectively,). Lesion volumes were similar for 12,000, 14,000, and 16,000 pulses/pulse at 1450 ± 190, 1460 ± 160, and 1480 ± 80 mm^3^, respectively (p > 0.05).

#### Total sonication time

The 27-point cubical pattern (Fig 1) was sonicated 5 to 30 times, thus varying the total sonication time. Visual inspection of slices perpendicular to the beam axis revealed three distinct planes of nine-point lesions for both 137- and 274-second long sonications. Longer total sonication times resulted in lesions that resembled a single large void. The entire lesion had a ‘tadpole’ shape along the beam axis for all total sonication times. Across the range of sonication times, lesion volumes increased linearly (R^2^ = 0.74) from 1020 ± 140 mm^3^ to 1490 ± 230 mm^3^, though without significant differences (overall ANOVA, p = 0.076, Fig 3c).

#### Pulse Repetition Frequency (PRF)

With all other sonication parameters kept constant, measurements of lesion volume exhibited significant differences with varying PRF (ANOVA p ≤ 0.0001, Fig 3d). Following sonications at 4 and 5 Hz PRF, the lesion head consisted of liquefied phantom debris. Lesion volumes increased linearly (R^2^ = 0.97) with increasing PRF. Pairwise comparisons reveal similar lesion volumes for 0.5 and 1 Hz, 0.5 and 2 Hz, 1 and 2 Hz, 2 and 3 Hz, and between 3 and 4 Hz PRF (p > 0.05). All other lesion volume comparisons resulted in significant differences (p ≤ 0.05), as seen in Fig 3d.

### Effect of BH sonication parameters on temperature elevation

Understanding temperature changes at or near the focal region provides insight on the effect of varying BH sonication parameters. Three coronal slices and one sagittal slice centered on the focal region were used to measure temperature changes relative to baseline during each sonication. Slices along both orientations were compared for each sonication. Additionally, temperature elevation for two different sonications (e.g., PRF of 0.5 and 5 Hz) was compared. FWHM was calculated from the temperature maps as a measure of temperature distribution for all sonication parameters.

#### Comparing temperature dynamics at different locations at the focal zone

**Fig 4:**
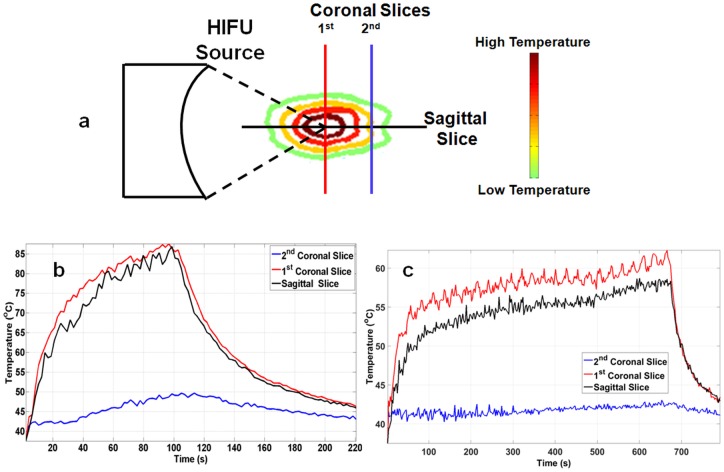
**a**. Illustration of temperature contours due to heat accumulating at focus. The position of coronal and sagittal MRI thermometry slices at which real-time temperature measurements are made, is also marked. **b**. Sonication performed at 600 W of acoustic power, 4 Hz PRF, and 15,000 cycles/pulse. Sagittal and first coronal slices had similar characteristics, reaching a peak temperature of 87°C. The second coronal slice had a maximum temperature of 46°C, throughout the entire sonication. **c**. Sonication performed at 600 W of acoustic power, at 1 Hz PRF for 684 seconds. Both first coronal and sagittal slices had similar temperature curve shapes, while the second coronal slice has marginal change in temperature, with a peak temperature of 63°C, 57°C, and 43°C respectively.

Fig 4a shows a comparison of temperature in first coronal (placed at the center of focus), second coronal (placed 7 mm from the center of focus), and sagittal slices for a sonication at 4 Hz PRF, 15,000 cycles/pulse, and 600 W acoustic power. The first coronal slice shows a rapid exponential increase in temperature until 100 seconds. At the end of sonication, a peak temperature of 87°C was attained. The sagittal slice displayed a similar peak temperature and trend in temperature. Post sonication, the temperature in both slices exponentially dropped to 46°C within 120 seconds. The second coronal slice demonstrated minimal temperature increase when compared to first coronal and sagittal slices. The maximum temperature in the second coronal slice was 49°C. Fig 4b shows a temperature comparison for all slices for a total sonication time of 684 seconds at 1Hz PRF and 600 W acoustic power. The first coronal slice indicated a rapid exponential temperature increase until 100 seconds; a similar result as observed in Fig 4a. Between 100 and 684 seconds, the temperature continued to increase, but at a lower rate. At the end of sonication, the peak temperature was 63°C. The sagittal slice showed a similar trend in observed temperature; a peak temperature of 57°C. Post sonication, the temperature within the ROI exponentially dropped to 43°C within 120 seconds. The second coronal slice in this case also displayed a minimal temperature increase when compared to first coronal and sagittal slices. The maximum temperature in the second coronal slice was 43°C.

#### Effect of sonication parameters on focal temperature dynamics

**Fig 5:**
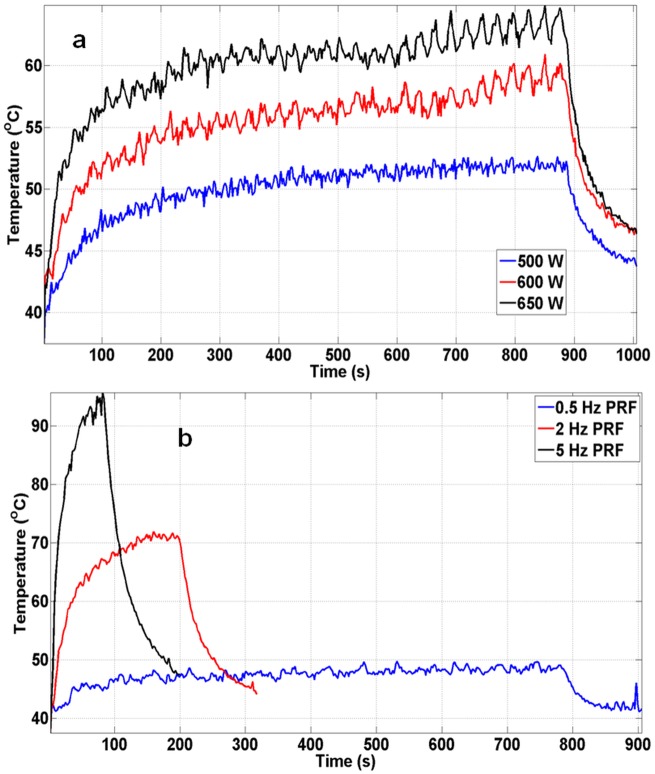
First coronal temperature slice was compared against first coronal temperature slices at different sonication parameters. **a**. Maximum temperature in tissue-mimicking phantoms compared at 500, 600, and 650 W of acoustic power. All three power settings produced similar exponential temperature increase and decrease, with different peak temperatures. **b**. Temperature curves obtained while sonicating at 0.5, 2, and 5 Hz PRF. The peak temperatures obtained were 50°C, 72°C, and 95°C for PRF of 0.5, 2, and 5 Hz, respectively.

Temperature curves from the first coronal slice were compared for acoustic powers of 550, 600, and 650 W, with other parameters kept constant (Fig 5a). All three curves indicated different rates of temperature increase, with temperatures plateauing at 52°C, 57°C, and 63°C respectively, after 200 seconds of sonication. Peak temperature was attained towards the end of the sonication and observed to be 53°C for 500 W, 60°C for 600 W, and 65°C for 650 W. A similar result is seen in Fig 5b for temperature comparison for sonications at 0.5, 2, and 5 Hz PRF, keeping other parameters constant. Temperature did not increase beyond 50°C at 0.5 Hz PRF. In addition, the rate of temperature increase between the three PRF values was different. Peak temperatures of 49°C, 72°C, and 95°C for 0.5, 2, and 5 Hz, respectively, were obtained. Peak temperature for 2 and 5 Hz PRF was attained in less than 70 and 150 seconds respectively.

#### Spatial temperature distribution at different sonication parameters

**Fig 6:**
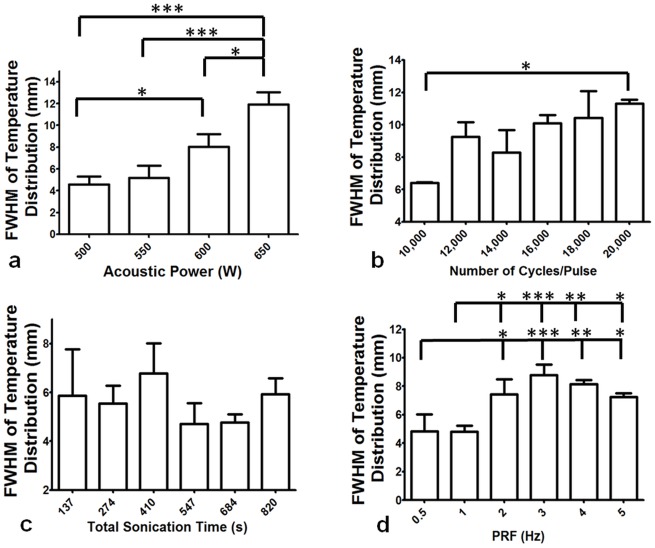
Full Width at Half Maximum (FWHM) was calculated as a measure of temperature distribution at the end of each sonication in tissue-mimicking phantoms. **a**. Varying acoustic power, significant differences in FWHM were found between 550 and 650 W, as well as for 600 and 650 W (p≤0.05). **b**. Comparing the number of cycles/pulse, significant differences were found between 10,000 cycles/pulse and 20,000 cycles/pulse (p = 0.042). Other parameters showed no differences in FHWM. **c**. Total sonication was varied from 137 to 820 seconds and no significant difference was found in the FWHM between any parameters (p > 0.05). **d**. Varying PRF from 0.5 Hz to 5 Hz resulted in significant differences in FWHM between 0.5 and 2, 3, 4, and 5 Hz. Similarly, significant differences were found between 1 and 2, 3, 4, and 5 Hz (p ≤ 0.05). No significant differences were found between any other parameters.

Exponential increase in temperature FWHM with increasing acoustic power was observed (R^2^ = 0.95). Significant differences in FWHM across all acoustic power values were found (overall ANOVA p ≤ 0.0001). Post-hoc test revealed no differences in FWHM between 500 and 550W, or between 550 and 600W (p > 0.05). All other acoustic powers were significantly different from each other (p ≤ 0.05, Fig 6a) in terms of temperature FWHM. While comparing FWHM across the number of cycles/pulse parameter, there was a linear increase in FWHM (R^2^ = 0.82), with overall significant differences (overall ANOVA p = 0.048). Significant difference between 10,000 and 20,000 cycles/pulse were found using post-hoc test (p ≤ 0.05). However, there was no significant difference in FWHM between any other parameters as observed in Fig 6b. Fig 6c shows that while extending total sonication time from 137 to 820 seconds and keeping other parameters constant, no significant difference was found in the FWHM between any parameters (p = 0.235). Exponential increase in FWHM was also observed with increasing PRF (R^2^ = 0.80). Significant differences were found across PRF values (overall ANOVA p = 0.0001). Post-hoc tests revealed no differences between 2 and 3, 4 or, 5 Hz nor between 3 and 4 or 5 Hz (p > 0.05). All other comparisons presented significant differences (p ≤ 0.05, Fig 6d).

#### Area of lethal thermal dose

**Fig 7:**
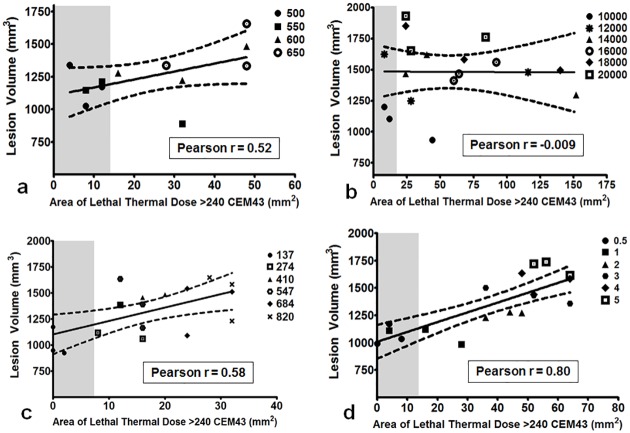
Correlation between lesion volume and area of lethal thermal dose greater than 240 CEM43. Grey area corresponds to 13 mm^2^, which is the area of each sonication layer. Any data point within this gray area corresponds to sonication parameters that produce thermal dose in an area equal to or less than the target area (13 mm^2^). **a**. Positive, insignificant correlation (Pearson r = 0.52, p = 0.08) was found between the two variables while varying acoustic power. **b**. No correlation was found while varying the number of cycles/pulse (Pearson r = -0.009, p = 0.97). **c**. Positive, strong correlation was found while varying total sonication time (Pearson r = 0.58, p≤0.001). **d**. Correlation between lesion volume and area of lethal thermal dose while varying PRF was also significantly positive (Pearson r = 0.80, p≤0.001).

Correlations between lesion volume and area of thermal dose provided insight to the relationship between these two variables based on varying sonication parameters. Acoustic power and area of lethal thermal dose > 240 CEM43 had a positive (Pearson r = 0.52) but insignificant correlation (p = 0.087, Fig 7a). Area of lethal thermal dose did not correlate with lesion volume for the number of cycles/pulse parameter (p = 0.97; Pearson r = -0.009), as seen in Fig 7b. However, total sonication time and PRF correlated well with lesion volume; the Pearson r was 0.58 and 0.80 with p = 0.012 and p ≤ 0.0001, respectively for both sonication parameters (Fig 7c & 7d).

### Boiling histotripsy lesions in *ex vivo* porcine tissues

**Fig 8:**
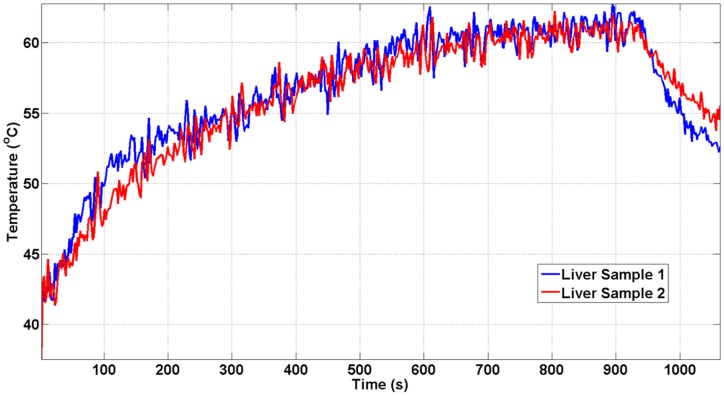
Temperature change measured at the first coronal slice for separate liver samples. The sonication parameters for both liver samples were 650 W of acoustic power, 15,000 cycles/pulse at 1 Hz PRF. The peak temperature was reached at the end of the sonication (64°C). Both temperature curves show similar dynamics.

**Fig 9:**
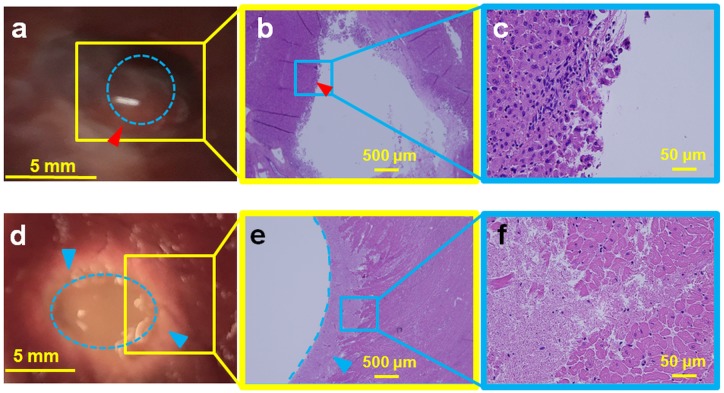
Ex vivo porcine liver (top panel) and cardiac muscle (bottom panel) sonicated at 600 W acoustic power at 1 Hz PRF and 20,000 cycles/pulse. **a**. Gross pathology of the liver tissue with the lesion in the center (red arrowhead), showing minimal thermal damage with a liquefied central void (blue dotted circle). **b**. H&E slide showing the entire lesion with sharp boundaries (red arrowhead). **c**. Magnification (4X) of the Fig 9b, presenting intact cell structures at the periphery of the lesion. **d**. Gross pathology of the cardiac tissue with a large void in the center (blue dotted oval). A concentric ring of necrosis (blue arrowheads) surrounds the central void. **e**. H&E slide, with the void outlined by the blue dotted line and the blue arrowhead pointing to the region of necrosis. **f**. Magnified image (40X) of Fig 9e show regions of both necrosis and intact cellular structures.

**Fig 10:**
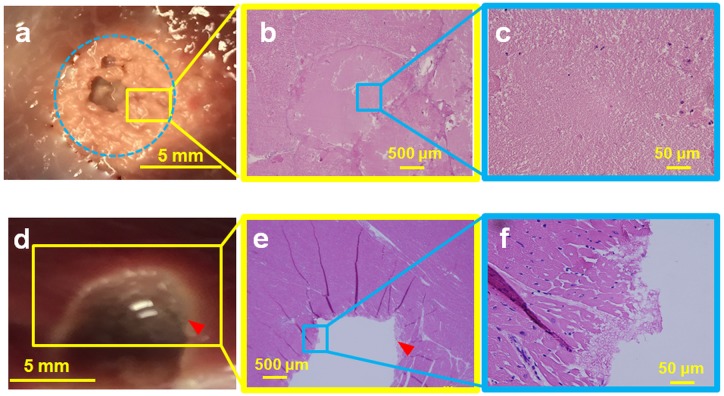
Ex vivo porcine liver (top panel) and cardiac muscle tissue (bottom panel) sonicated with 600 W at 5 Hz PRF. **Top Panel: a**. Gross pathology of the lesion appeared thermally denatured, with a diameter of 8 mm (blue dotted circle). This area was not structurally intact and disintegrated during pathological stain preparation process. **b**. H&E slide showing diffuse thermal effects, with little cellular structure. **c**. Magnification (40X) of the Fig 10b with parts of the lesion showing absence of cellular structure, representing homogenization of tissue. **Bottom Panel. d**. Cardiac muscle with a liquefied void in the center of the lesion with sharp boundary with negligible thermal effects (red arrowhead). **e**. H&E slide of the lesion shows that cellular structure around the void was intact, with no evidence of thermal damage. The lesion also has sharp boundaries (red arrowhead). **f**. Magnification (40X) of the lesion boundary, with most of the boundary tissue intact.

**Fig 11:**
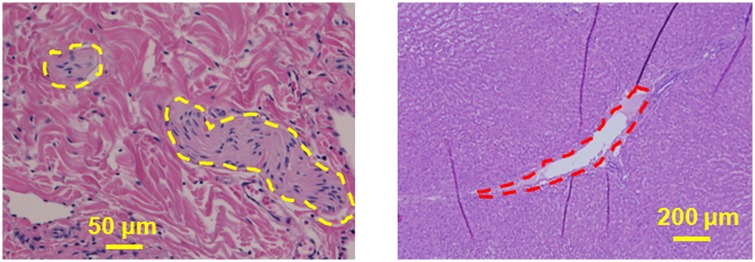
Liver tissue was sonicated with 650 W at 1 Hz PRF. **a**. Dotted yellow margins represent the nerves in liver tissue that were intact post sonication. These nerves were situated less than 300 μm from the focal region, showing the ability of BH to spare nerves. **b**. Bile ducts located less than 500 μm from the focal region were also structurally intact (red dotted margin) post sonication. Tissue surrounding the bile duct was also intact and did not have any signs of necrosis.

We additionally demonstrated the ability of this clinical MR-HIFU system to perform BH in *ex vivo* tissues, with the ultimate intent to apply BH *in vivo*. Temperature data were similar between two consecutive liver sonications at 600 W acoustic power at 1 Hz PRF (Fig 8a). The post sonication H&E images are shown alongside the corresponding gross pathology photographs in Figs 9, 10 and 11. Gross morphological analysis showed similar lesions for sonications repeated with same parameters. Differences in qualitative morphological parameters such as lesion shape and size were observed between sonication at 1 Hz PRF, 600 W, and 20,000 cycles/pulse and sonication at 5 Hz PRF, 600 W, and 15,000 cycles/pulse.

Sonicating porcine liver with 20,000 cycles/pulse, 600 W acoustic power at 1 Hz PRF, formed a large lesion with its contents completely liquefied, as observed in top panel of Fig 9. The lesion had sharp boundaries with thermal damage (red arrowhead, Fig 9a). The corresponding H&E slide shows negligible necrosis on one side, while there is evidence of necrosis on the opposite side (Fig 9b & 9c). Closer examination of the H&E slide reveals the necrotic region to be less than 400 microns in diameter, with a sharp lesion boundary (red arrowhead, Fig 9b & 9c). Critical structures such as bile ducts (red dotted margin) and nerves (yellow dotted margin) appeared physically intact 2 mm away from the focal region (Fig 11). Fig 9d shows a photograph of sonicated cardiac tissue with an 8 mm diameter lesion filled with liquefied tissue (blue dotted circle). The lesion had a white concentric band, possibly due to thermal denaturation. The cardiac tissue H&E slide shows a corresponding necrotic band of tissue around the lesion with a sharp boundary, similar to the liver tissue (Fig 9e & 9f).

To determine the effect of higher PRF at 15,000 cycles/pulse and 600 W acoustic power, porcine liver was sonicated using these parameters at 5 Hz PRF. The resultant liver lesion had a whitish, disc shaped region, 8 mm in diameter (Fig 10a). This region appeared thermally denatured with no structural integrity. While preparing the tissue for H&E stain, the thermally denatured region at times disintegrated into paste-like debris, leaving behind a hole. H&E slides show tissue completely fractionated with no intact cells at the focal region (Fig 10b & 10c). Fig 10d shows the gross pathology of cardiac tissue, displaying a hole at the center of the focal region filled with liquefied tissue debris. The boundary of this lesion has a thin white rim, possibly providing some evidence of marginal thermal denaturation (red arrowhead). The corresponding H&E stain shows a hole with a sharp boundary (red arrowhead, Fig 10e). The boundary also contains a small necrotic region, while the rest of the tissue appears intact with no signs of necrosis, as seen in the magnified image (Fig 10f).

### MRI monitoring of *ex vivo* tissue destruction

**Fig 12:**
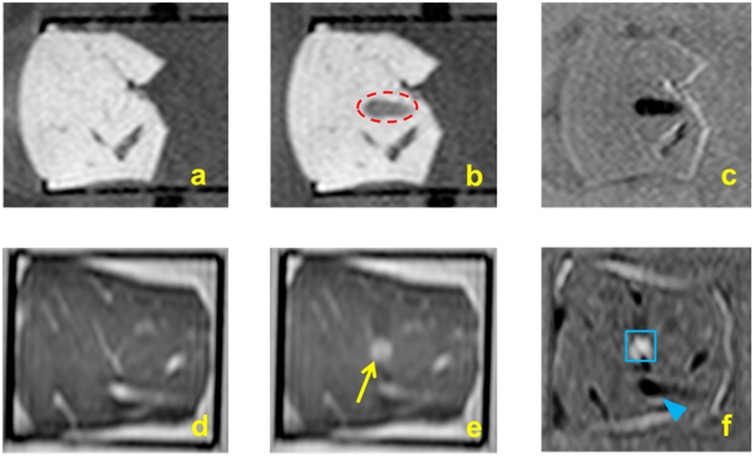
**a**. T1W imaging of porcine liver using fast field echo 3D used for planning HIFU sonications **b**. T1W imaging of the liver tissue post sonication shows a hypointense signal of a tadpole-shaped lesion created using BH (red dotted circle) **c**. Subtraction of pre-sonication from post-sonication (Fig 12b minus Fig 12a) images highlights the BH lesion. **d**. T2W imaging using turbo spin echo 3D were also performed **e**. T2W imaging post sonication showing the BH lesion along the coronal plane **f**. Subtraction of pre-sonication from post-sonication images (Fig 12e minus Fig 12d) highlighting the lesion (blue box) and surrounding blood vessels (blue arrowhead).

Post sonication T1W imaging of the liver after BH revealed a hypointense region (red dotted circle, Fig 12b). Both the lesion boundaries and the tail of the lesion are clearly detectable (Fig 12c). Fig 12e shows a post-sonication T2W coronal image with a visible lesion (yellow arrow). The subtraction image of pre- and post-sonication images shown in Fig 12f demonstrates the lesion from top view (blue box) and adjacent blood vessels (blue arrowhead).

## Discussion

This experimental work focuses on characterization of the effect of BH sonication parameters on resulting lesion volume, temperature distribution, and thermal dose. Such characterization is necessary to define the extent of BH lesion formation with mechanical damage, thermal effect, or a combination of both. Thermal and mechanical effects in tissue-mimicking phantoms and *ex vivo* tissue due to BH have been briefly described by Khokhlova *et al* [56]. In order to characterize the effect of several additional BH sonication parameters such as acoustic power, number of cycles/pulse, total sonication time, and PRF on lesion characteristics, we examined lesion volume, temperature distribution, and thermal dose.

The current experimental work produced lesion volumes greater than 1 cm^3^ both in TMP’s and *ex-vivo* tissues, suggesting that a clinical MR-HIFU system can produce large, clinically relevant BH lesion volumes. Table 3 provides a synopsis of the effect of sonication parameters on lesion volume, temperature distribution and area of lethal thermal dose. Considering that the ‘head’ part of the tadpole-shaped lesion is the largest contributor to lesion volume, the segmentation results did not show a significant change with increasing acoustic power. Comparison of pulse length between 10,000 cycles/pulse to 18,000 cycles/pulse and, 20,000 cycles/pulse did however increase lesion volume. This is due to increase in energy deposition, causing greater damage to the tissue-mimicking phantom. Our results also show that if pulse length, power and PRF are kept constant, the lesion volume does not vary much with increasing total sonication time.

**Table 3:** Effect of each of the four BH sonication parameters on significant increase in lesion volume and focal region temperature are summarized. Increasing acoustic power did not result in an increase in lesion volume, whereas temperature distribution and area of lethal thermal dose increased. While increasing number of cycles/pulse, both lesion volume and temperature distribution increased. No increase in either lesion volume or temperature distribution was observed while increasing total sonication time, but area of lethal thermal dose increased. Varying pulse repetition frequency resulted in increased lesion volume and temperature distribution and area of lethal thermal dose.

Parameter Increase	Lesion Volume Increase	Focal Temperature Distribution Increase	Thermal dose area at 240 min (CEM43) Increase
Acoustic Power	No	Yes	Yes
Number of Cycles/Pulse	Yes	Yes	No
Total Sonication Time	No	No	Yes
PRF	Yes	Yes	Yes

Increasing acoustic power produced a significant increase in temperature distribution. This could be due to increased incident acoustic pressure reducing the time-to-boil causing quicker and enhanced heating at the focal region. In addition, the lack of perfusion complements this effect in both tissue mimicking phantoms and *ex-vivo* tissues. Thus, this model, using the weak shock theory to calculate time-to-boil, may not perfectly represent changes *in vivo* tissue. To obtain the time-to-boil more accurately for *in vivo* experiments, a model that considers diffusion effects needs to be explored in the future. A similar difference in temperature distribution between 10,000 cycles/pulse and 20,000 cycles/pulse was observed, likely due to an increase in total energy deposited which is known to increase both focal region temperature and lesion volume. Increase in total sonication time shows that there is a threshold total sonication time or number of pulses per location, beyond which lesion volume or temperature no longer increase. Prior studies have shown an increase in PRF or DC causing an increase in rate of energy deposition at the focal region [56]. This is also reflected in our results with a significant increase in temperature FWHM and lesion volume while increasing PRF.

Correlation between lesion volume and area of lethal thermal dose provided valuable information on the effect of sonication parameters on tissue-mimicking phantoms. Our experiments show that for both 500 and 550 W acoustic powers, the lethal thermal dose was contained within the targeted sonication area (13 mm^2^, grey region in Fig 6). Although strong correlation was not found between area of lethal thermal dose and lesion volume while varying number of cycles/pulse, there is some evidence that 20,000 cycles/pulse could produce larger lesion volumes than 10,000 cycles/pulse yet yield a similar area of lethal thermal dose. This could have valuable clinical implications and may need to be studied further. Lower total sonication times and PRF values contained the lethal thermal dose within the targeted sonication area. Depending on the application and the target location, Table 3 and Figs 2, 5 and 6 provide a basic pathway in selecting sonication parameters for future BH studies.

The relatively low spatiotemporal resolution of MRI thermometry may result in peak temperature measurement inaccuracies when monitoring boiling that occurs on the order of milliseconds. However, unlike in HIFU thermal ablation, macroscopic temperature changes as produced by the BH technique are slow due to low applied duty cycles. MRI provides a measurement of the mean temperature within a voxel. However, the peak intra-voxel temperature can be higher, depending on the spatial acquisition resolution. In this study, the size of the focal point (1.6 × 1.6 × 10 mm) was smaller than the MRI voxel size (2.5 × 2.5 × 7 mm), which can lead to inaccuracies in temperature and thermal dose measurements. While the Sonalleve therapy planning software compensates for temperature standard deviation in thermal dose calculations, it does not compensate for intra-voxel spatial temperature variations. This does not pose a substantial issue in our study, however, since we only compare the differences in temperature and thermal dose with varying sonication parameters; not accurately characterize the cumulative thermal dose.

To relate to clinical HIFU therapy, we placed three MRI thermometry slices along the coronal plane and one slice along the sagittal plane, to monitor temperature related effects throughout the focal region. As expected, the temperature-time series data at the sagittal plane was similar to the temperature-time series data from the first coronal slice, which was at the middle of the sonication zone. Temperature measured at the second coronal slice for all sonication parameters was well under 50°C, explained by the fact that this slice was 7 mm away from the focus. This result could be influential in future *in vivo* applications, including local temperature-triggered, tumor specific drug release, immunomodulation and other bio-effects. Temperature curves for sonications performed at 1 Hz PRF had a characteristic cyclic variation in temperature that lasted for 27 seconds. This follows the sonication pattern where the 27-point cube is sonicated at one location per second. As the sonication proceeded, the bottom and middle layer (nearest to the transducer) broke down first causing a drop in temperatures. The top layer (farthest from transducer) and phantom material beyond was still intact and therefore the temperature continued to rise. The other factor that may have caused this effect is the location of the sonication layer being farther away from the center of the temperature-mapping slice. This causes the measured temperatures to be slightly lower than the temperature measured at the center of the temperature-mapping slice.

*Ex vivo* tissues were sonicated to demonstrate the capability of this clinical MR-HIFU system to perform BH *in vivo*. Porcine liver and cardiac muscle that were sonicated with identical parameters yielded lesions that were visually different. Lesion in the liver tissue appeared to be larger, with greater tissue destruction compared to the cardiac muscle, possibly attributable to the differing mechanical properties of the tissues. At 600 W, 1 Hz PRF, and 20,000 cycles/pulse, the liver tissue was mechanically disrupted, with a large visible void at the focal region. The lesion had some thermal effects on one side of the lesion, possibly due to increased thermal dose at that region. For the same set of sonication parameters, the cardiac tissue had a hole at the center of the focal region surrounded by concentric ring of tissue necrosis around the lesion (Fig 9). The concentric ring was due to uniform heating at the middle of the ‘head’ of the BH lesion causing thermal damage to tissue. Sonicating liver tissue at 5 Hz PRF, 15,000 cycles/pulse, and 600 W, the focal region had a paste-like circular lesion, indicating significant thermal effects. In contrast, cardiac tissue at these parameters has a void filled with liquefied tissue. There was also no evidence of thermal denaturation beyond this void. This interesting finding shows the variability in lesion formation in different tissue types for the same BH sonication parameters. BH may therefore need to be characterized or calibrated on a tissue or organ specific basis. Additionally, our data show the ability of BH to retain adjacent tissue structure for long sonication periods, high acoustic powers, or PRFs (Fig 11). This finding is vital for future *in vivo* experiments since it shows the ability for vital vessel- or structure-sparing using a clinical HIFU system, thus potentially opening new preclinical and clinical avenues. In addition, the ability to visualize BH lesions on MRI indicates that an MRI contrast agent may not be required *in vivo*. However, MRI contrast agents used *in vivo* may further benefit MR imaging of BH lesions post sonication, which needs to be explored further.

## Conclusion

This experimental work characterizes and quantifies the effect of varying BH sonication parameters using a commercially available clinical MR-HIFU system. The effects quantified include temperature, thermal dose, and lesion volume. Experiments were performed utilizing tissue-mimicking phantoms and *ex vivo* tissues. Results indicate varying lesion volumes and temperature effects for different BH sonication parameters. Future work may need to characterize BH effects *in vivo* in different tissues and organs. Such a characterization may facilitate clinical translation of BH. Our results also provide parameter recommendations for future BH experiments. In the future, it may be useful to test the short- and long-term effects of varying these sonication parameters extensively *in vivo* using this MR-HIFU system. Additionally, it will be imperative to assess the effects of tissue perfusion on lesion formation, lesion volumes, and temperature elevation.

## Supporting information

S1 Data The supplemental table contains individual worksheet for lesion volume, thermal dose and temperature FWHM for all sonication parameters used in this experimental work. Each worksheet in this data file shows the values obtained for 3D segmented lesion volume, 240CEM43 and temperature FWHM for each sonication parameter varied in this work.
(XLSX)
